# The prediction of all-cause mortality in end-stage kidney disease patients using social determinants of health: A machine learning framework

**DOI:** 10.1016/j.imu.2026.101779

**Published:** 2026-06-19

**Authors:** Addy Smith, Maxwell Donelan, Hossein Moradi Rekabdarkolaee, Patti Brooks, Brandon M. Varilek, Surachat Ngorsuraches, Jerry Schrier, Adam Dell, Semhar Michael

**Affiliations:** a Department of Mathematics and Statistics, South Dakota State University, Brookings, SD, USA; b Bowling Green State University, Department of Business Analytics, Economics, and Information Systems, Bowling Green, OH, USA; c College of Business and Information Systems, Dakota State University, Madison, SD, USA; d College of Nursing, University of Nebraska Medical Center, Omaha, NE, USA; e Department of Health Outcomes Research and Policy, Auburn University, Auburn, AL, USA; f Avera Medical Group Nephrology, Avera McKennan Hospital & University Health Center, Sioux Falls, SD, USA; g Department of Pediatrics, University of South Dakota, Vermillion, SD, USA

**Keywords:** Machine learning, End-stage kidney disease, Mortality prediction, Public health, Social determinants of health

## Abstract

End-stage kidney disease (ESKD) is the irreversible final stage of chronic kidney disease in which the kidneys lose their independent function. This study presents a machine learning framework to predict all-cause mortality in ESKD patients by the end of a follow-up period. We combined patient-specific clinical factors with social determinants of health (SDOH) to assess their influence on survival outcomes. Data were obtained from the United States Renal Data System, including patients admitted in 2015 and followed through August 2021. Community-level SDOH data were integrated from the Agency for Healthcare Research and Quality, with variable screening techniques and expert input guiding feature selection. To address class imbalance, the synthetic minority oversampling technique (SMOTE) was applied. Three machine learning models were developed: logistic regression, random forest, and extreme gradient boosting. Model performance was assessed using accuracy, sensitivity, specificity, and area under the receiver operating characteristic curve (AUC). Model calibration was assessed using a calibration curve and a Brier score. The extreme gradient boosting model performed best, with an AUC of 0.7947, although other models showed comparable results. Including community-level SDOH features did not significantly improve model performance overall or within subpopulations. This suggests patient-level variables are the primary drivers of mortality prediction in ESKD. Furthermore, SMOTE did not enhance model performance in subpopulations.

## 1. Introduction

End-stage kidney disease (ESKD) is the final, irreversible stage of chronic kidney disease (CKD) in which the kidneys cannot function independently, thus requiring dialysis or a kidney transplant for survival. In 2023, over 800,000 residents of the United States lived with ESKD, according to the United States Renal Data System (USRDS) [1]. ESKD severely impacts a patient's quality of life, imposing both physical and emotional burdens. Dialysis is a demanding process that requires multiple sessions per week, while kidney transplants remain difficult to access due to long wait lists and limited donor availability. Despite advances in clinical management, ESKD patients continue to face disproportionately high mortality rates.

Previous research on predicting all-cause mortality in ESKD patients typically relied on regression-based models that focus on clinical and demographic covariates. However, such approaches may overlook the complex interplay of social determinants of health (SDOH) with medical variables in predicting patient survival outcomes. SDOH are the non-medical factors that influence patient health outcomes, relating to the conditions in which individuals are born, grow, work, live, worship, and age [2,3]. Examples include data on socioeconomic status, employment, education, housing, transportation, food access, and healthcare access. It is estimated that while clinical care impacts about 20 percent of health outcomes at the county level, SDOH factors can affect as much as 50 percent [4]. Incorporating a comprehensive set of SDOH alongside clinical predictors could offer a better understanding of the role that SDOH plays in determining mortality risks in patients with ESKD.

More recently, machine learning (ML) methods have been applied to kidney disease outcomes, including dialysis mortality prediction. Random forests and gradient boosting machines have demonstrated superior discrimination compared to logistic regression in mortality prediction [5,6]. Neural networks and ensemble learners have also been explored for short- and long-term mortality risk stratification in hemodialysis patients, often improving sensitivity at the expense of interpretability [7]. Comparative studies generally show that tree-based models (RF, XGBoost) achieve robust performance but often perform similarly to penalized regression when strong individual-level demographic and clinical predictors dominates. Time-to-event survival models have also been used to capture mortality disparities among individuals with ESKD [8,9]. However, these studies did not incorporate a comprehensive array of community-level social determinants of health (SDoH) variables.

A recurring challenge in predictive modeling is class imbalance. The Synthetic Minority Oversampling Technique (SMOTE) is one of the most widely applied remedies, generating synthetic samples of the minority class near decision boundaries [10]. Although SMOTE often improves classification performance in imbalanced datasets, evidence from clinical prediction studies indicates mixed benefits, with possible gains in specificity at the cost of calibration and sensitivity [11]. Another key methodological issue is feature selection. Most large-scale ESKD datasets, such as USRDS, contain structured demographic and clinical indicators but lack granular, longitudinal laboratory measurements as well as other SDOH. Therefore, many rely on community-levels proxies for SDOH as opposed to patient-level indicators. This in turn results in a large possibly highly correlated SDOH factors at community level. Although community-level measures are imperfect proxies, there is evidence that neighborhood resources, socioeconomic hardship, and healthcare access meaningfully influence outcomes ESKD, indicating a need to further explore the association between community-level measures and ESKD outcomes [12,13]. Consequently, with multitudes of community level health indicators, variable selection techniques such as LASSO regression are frequently employed to reduce dimensionality before ML training [14]. In addition, reliance on community-level proxies for SDOH, as opposed to patient-level indicators, may underestimate the true role of social context. Singh et al. suggests that SDOH effects are pronounced earlier in CKD progression, with diminished impact once patients reach advanced stages of disease [12]. This highlights the need for integrating longitudinal electronic health record (EHR) features, patient-reported SDOH measures, and continuous updates of comorbidities and lab values in future models. While recent advancements in ESKD mortality prediction have utilized deep learning architectures, such as transformers for longitudinal EMR data [15] or convolutional neural networks for renal microvascular imaging data [16], these models often require extensive amounts of data inputs to train which are not universally available (e,g., for each patient on USRDS database). Our study fills a distinct gap by focusing on the predictive ability of community-level SDOH when integrated with standard, structured registry data (USRDS). Unlike models optimized for clinical settings with imaging or temporal sequences, our framework evaluates whether widely accessible Zip-code/County-level non-medical factors can improve risk stratification in a stable pre-pandemic cohort, providing a benchmark for the marginal utility of non-clinical data. While advanced models such as XGBoost can capture nonlinear relationships and achieve modest improvements over traditional regression, patient-level clinical factors remain the strongest predictors, and methodological challenges, including imbalanced outcomes, limited individual-level SDOH data, and interpretability concerns, must be carefully addressed. By combining patient-specific clinical characteristics with community-level SDOH indicators and systematically evaluating oversampling methods, this study contributes to the growing field of ML-based mortality prediction in ESKD.

## 2. Materials and methods

### 2.1. Data sources

Data was obtained from two main sources: the United States Renal Data System (USRDS) [1] and the Agency for Healthcare Research and Quality Social Determinants of Health (AHRQ-SDOH) Database [17]. The USRDS dataset contained patient-level information on ESKD patients from a 2015 cohort, all of whom were de-identified. These patients were followed from 2015 until August 2021, at which point they were classified as “deceased or “not deceased.” All variables in the USRDS dataset were measured at the time of admission in 2015, except for the variable recording the patient's mortality status at the end of the follow-up period. The AHRQ-SDOH datasets contained community-level information on United States ZIP codes and counties. The database was an AHRQ project to collect and use SDOH-focused data from multiple sources. It was developed to make it easier to find a range of well documented, readily linkable SDOH variables across the SDOH domains without having to access multiple source files. The majority of information for the variables we used came from the American Community Survey (ACS) [17], which is data derived from the US Census Bureau. All variables in the AHRQ-SDOH datasets were measured during 2015 or 2020 and related to the social context, economic context, education, physical infrastructure, and healthcare context at the given community level.

### 2.2. Data cleaning

Several exclusion criteria were applied to prepare the datasets for modeling. For the USRDS dataset, patients over the age of 108, as well as those with unknown race, ethnicity, or rurality, were excluded. Patients missing information on primary disease, alcohol dependency, employment, insurance type, modality, or body mass index (BMI) were also removed from the dataset. Additionally, patients residing outside the 50 United States were excluded.

For the community-level AHRQ-SDOH datasets, ZIP codes and counties falling within U.S. territories or those having no population were removed from the datasets. Moreover, variables whose data was sensitive or private in such a way that data points below a certain threshold were missing by design to protect privacy. Other variables, such as those dealing with the percentage of subpopulation with a certain characteristic, were missing where the subpopulations were 0. For these cases, the percentage of variables were changed to total population variables so that the missing values could be replaced with zero indicating no individuals of the subpopulation with that characteristics were observed. Additionally, all variables containing 50% or more missing values were removed. For the remaining missing values in the community-level datasets, the values were imputed using the predictive mean matching method implemented in the R package MICE [18]. After cleaning was performed on the USRDS and AHRQ-SDOH datasets separately, they were merged into a new dataset by linking the ZIP code and county FIPS code associated with each patient. Finally, all categorical variables were converted into numeric variables to ensure compatibility with the ML techniques that required numeric data. This was done through one-hot encoding.

### 2.3. Community and individual-level variable selection

**Fig. 1.**
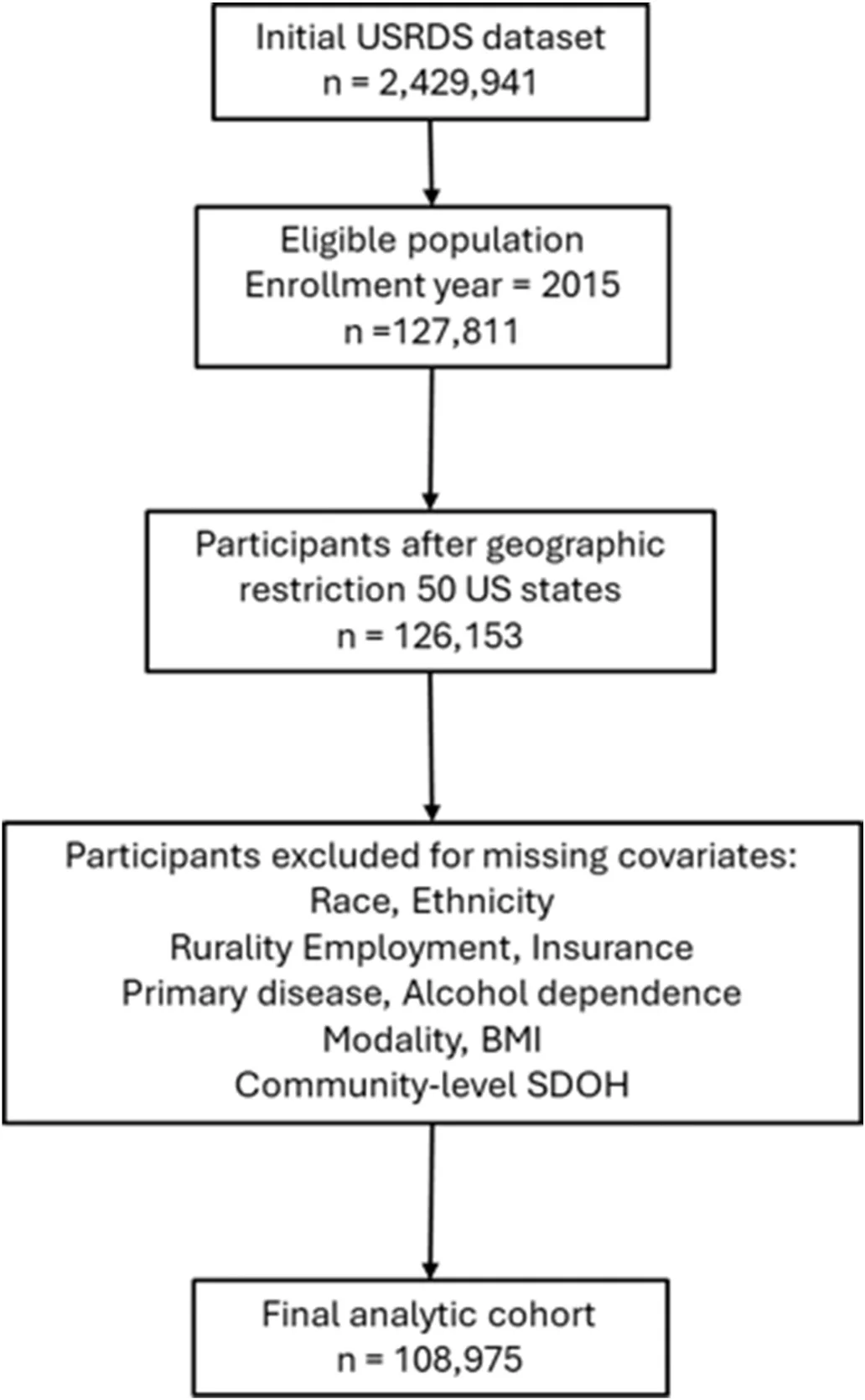
USRDS and Community level data for final cohort selection.

Both expert review and a statistical approach were used to reduce the initial set of over 300 community-level SDOH variables (excluding demographic variables) to a more manageable number, retaining only those most relevant for modeling. The list of potential SDOH predictor variables was reviewed by three collaborators with backgrounds in health and informatics. Each collaborator rated the variables on a scale from zero to three. A rating of zero indicated that the variable was a sociodemographic and should be excluded from the final set of SDOH variables retained. A rating of one signified low importance SDOH, two indicated moderate importance SDOH, and three represented high importance SDOH. Variables that received ratings of two or higher from all collaborators were retained for further analysis. SDOH variables were also screened using Sure Independence Screening (SIS), via the R package SIS [19]. The SDOH variables were screened using ESKD risk scores calculated at the community level to determine the most important SDOH variables for predicting ESKD mortality [20]. Variations on the SIS model were tested, and the SDOH variables were ranked by how often they were chosen by the model. This was combined with the expert review to form a subset of the SDOH variables containing only the most important variables. The remaining set of SDOH variables, along with all patient-level variables, were further filtered using Least Absolute Shrinkage and Selection Operator (LASSO) regression [14]. In this approach, all remaining candidate variables were used as predictors, and those with coefficient estimates shrunk to zero were excluded from the final set. The selected variables were then used as inputs in the development of our machine learning models. One limitation of using LASSO regression for feature selection, however, is its underlying assumption of linearity, which may not fully capture complex relationships among variables. After variable selection, the final dataset used for modeling consisted of 108,975 rows, with one column representing the target variable, “Death by End of Follow-Up Period.” The final cohort selection diagram is presented in Fig. 1. The full characteristics table of the data is shown in Appendix Table A.2. Furthermore, to address potential selection bias inherent in our complete-case approach, we conducted a comparative analysis of the included (n = 108,975) and excluded cohorts. Baseline characteristics, including age, sex, and primary disease, remained consistent across both groups (see Appendix Table A.3), supporting the assumption that data were missing at random and that the exclusion criteria did not fundamentally alter the cohort's clinical profile.

### 2.4. Machine learning algorithms

Three ML models were used to predict whether a patient would die by the end of the follow up period, namely logistic regression (LR) [21], random forest (RF) [22], and extreme gradient boosting (XGB) [15] Hyperparameter tuning was completed for the random forest and extreme gradient boosting models using 10-fold cross-validation and a grid search approach. Model performance was assessed using 10-fold cross-validation and four performance metrics: area under the receiver operating characteristic curve (AUC), accuracy, sensitivity, and specificity. AUC was the primary metric used to evaluate model performance. In addition to discriminative metrics (AUC), we assessed model calibration to ensure that predicted probabilities accurately reflect observed mortality risks. Calibration was evaluated via Brier scores and visually inspected via calibration plots comparing predicted vs observed risk deciles.

### 2.5. Oversampling algorithm

Our dataset was imbalanced, with more patients who died by the end of the follow-up period than those who survived. Additionally, the majority of patients were White or non-Hispanic, with fewer individuals from minority racial and ethnic groups. To address this imbalance, we applied the synthetic minority oversampling technique (SMOTE) [10,11], a common method used in similar research to generate synthetic samples of the minority class of the target variable. The algorithmic process for applying SMOTE to the model training data is outlined in Algorithm 1.

### 2.6. Software used

The analysis of this study was conducted using R version 4.4.1. From the R software, the algorithms implemented used the following libraries: glmnet (LASSO regression), stats (logistic regression), randomForest (random for-est), xgboost (extreme gradient boosting), and themis (SMOTE algorithm).

**Algorithm 1.** SMOTE with k-fold cross-validation

**Require:** Dataset D = (**X**, y), Number of folds *K*, Classifier M	
**Ensure:** Performance metrics (AUC, accuracy, sensitivity, specificity)	
1:	Initialize *K*-fold stratified cross-validation
2:	Initialize lists to store performance metrics
3:	**for** each fold i = 1, 2, …, K **do**
4:	Split D into training set *D*_train_ and test set *D*_test_
5:	Stratify *D*_train_ by race and ethnicity
6:	Apply SMOTE within each strata of *D*_train_ to generate synthetic samples
7:	Train classifier M on the balanced *D*_train_
8:	Evaluate M on *D*_test_
9:	Store performance metrics
10:	**end for**
11:	Compute average performance metrics over all k folds
12:	**return** AUC, accuracy, sensitivity, and specificity

## 3. Results

The following section compares the performance of the three proposed ML modeling techniques using different combinations of patient-level variables and community-level SDOH variables. The performance of the models is also compared when trained on the original data versus the oversampled data. Additionally, the importance of the variables used as predictors during modeling is assessed to identify which variables have the greatest influence on predicting all-cause mortality in ESKD patients. We then make inference about the directionality of the predictor variables in relation to the target variable using the logistic regression model.

### 3.1. Model performance using 10-fold cross validation

To explore the role of SDOH in predicting all-cause mortality in ESKD patients, we tested various combinations of patient- and community-level SDOH variables across the three proposed ML modeling techniques. The goal was to assess how these combinations impact overall model performance and performance within specific racial and ethnic subpopulations. We analyzed four different variable combinations that were tested in the models: 1) the 59 community-level SDOH variables obtained through variable screening; 2) all patient-level variables, excluding race and ethnicity; 3) all patient-level variables; 4) all patient-level variables plus the 59 community-level SDOH variables. A list and definitions of the USRDS patient-level and AHRQ-SDOH community-level variables are included in Appendix Table A.1. The models discussed in this subsection were not trained on oversampled data. The overall performance metrics for each model type and variable combination are summarized in Table 1. The highest AUC was observed with the extreme gradient boosting model that included all patient-level variables, achieving an AUC of 0.7947. This model also achieved the highest accuracy at 0.7424. The full results of the performance metrics including sensitivity and specificity are shown in Appendix Table A.4. Its sensitivity and specificity were 0.8623 and 0.5276, respectively. When comparing the best-performing model (highest AUC) for each ML approach, the three models displayed similar performance metrics. The best-performing logistic regression model, which included all patient-level variables and community-level SDOH variables, achieved an AUC of 0.7923, accuracy of 0.7394, sensitivity of 0.8560, and specificity of 0.5305. The best-performing random forest model, which included all patient-level variables, achieved an AUC of 0.7823, accuracy of 0.7383, sensitivity of 0.8548, and specificity of 0.5296. When comparing the best-performing models across the three ML approaches, only minor differences in performance metrics were observed, highlighting the overall consistency in predictive ability across the models. To contextualize model performance, we compared our approach against two established clinical baselines: a comorbidity-only model (AUC = 0.6791) and a comorbidity-plus-age model (AUC = 0.764). Our final model achieved an AUC of 0.7947, representing an 11% and 3% relative improvement, respectively. These differences are meaningful given the cohort size (>100,000 patients). These comparisons help situate our model within clinically relevant benchmarks and clarify the incremental value gained beyond existing risk scores. Another key finding from the results in Table 1 was the comparison of model performance via AUC using only patient-level variables versus models that also included the 59 community-level SDOH variables. Quantitatively, the inclusion of the community-level SDOH variables led to the following changes: an increase in AUC by 0.0009 for the logistic regression model, a decrease in AUC by 0.0003 for the random forest model, and a decrease in AUC by 0.0005 for the extreme gradient boosting model. Further, when examining the performance of models using community-level SDOH variables only, the AUC values were substantially lower: 0.5749 for logistic regression, 0.5432 for random forest, and 0.5848 for extreme gradient boosting. Since an AUC of 0.5000 indicates no ability to distinguish between classes of the target variable, our results suggested that models using only community-level SDOH variables have minimal predictive ability. This can be seen by the estimated 95% confidence intervals for the difference in mean AUC between the model with and without SDOH given by (−0.0149, 0.0131) for logistic regression, (−0.0168, 0.0174) for random forest, and (−0.0141, 0.0151) for the extreme gradient boosting model. We also found that the addition of community-level SDOH variables as predictors into our models did not substantially improve model performance within racial, ethnic, rurality, and region subpopulations. We propose two possible reasons for the limited predictive ability of community-level SDOH. One reason could be that the SDOH used in this research were measured at the community level, rather than being specific to individual patients. Additionally, the aggressive and rapidly progressing nature of ESKD may limit the role of SDOH in predicting mortality at this advanced stage. Our findings align with those of Singh et al. [12], who observed that SDOH played a smaller role than patient-level factors in predicting outcomes for patients with known CKD. The authors suggested that SDOH may have a more significant impact during the earlier stages of the disease. For patients in the end stages where the disease has progressed significantly, SDOH might have a diminished effect on risk prediction.

### 3.2. Use of oversampling algorithms

**Fig. 2.**
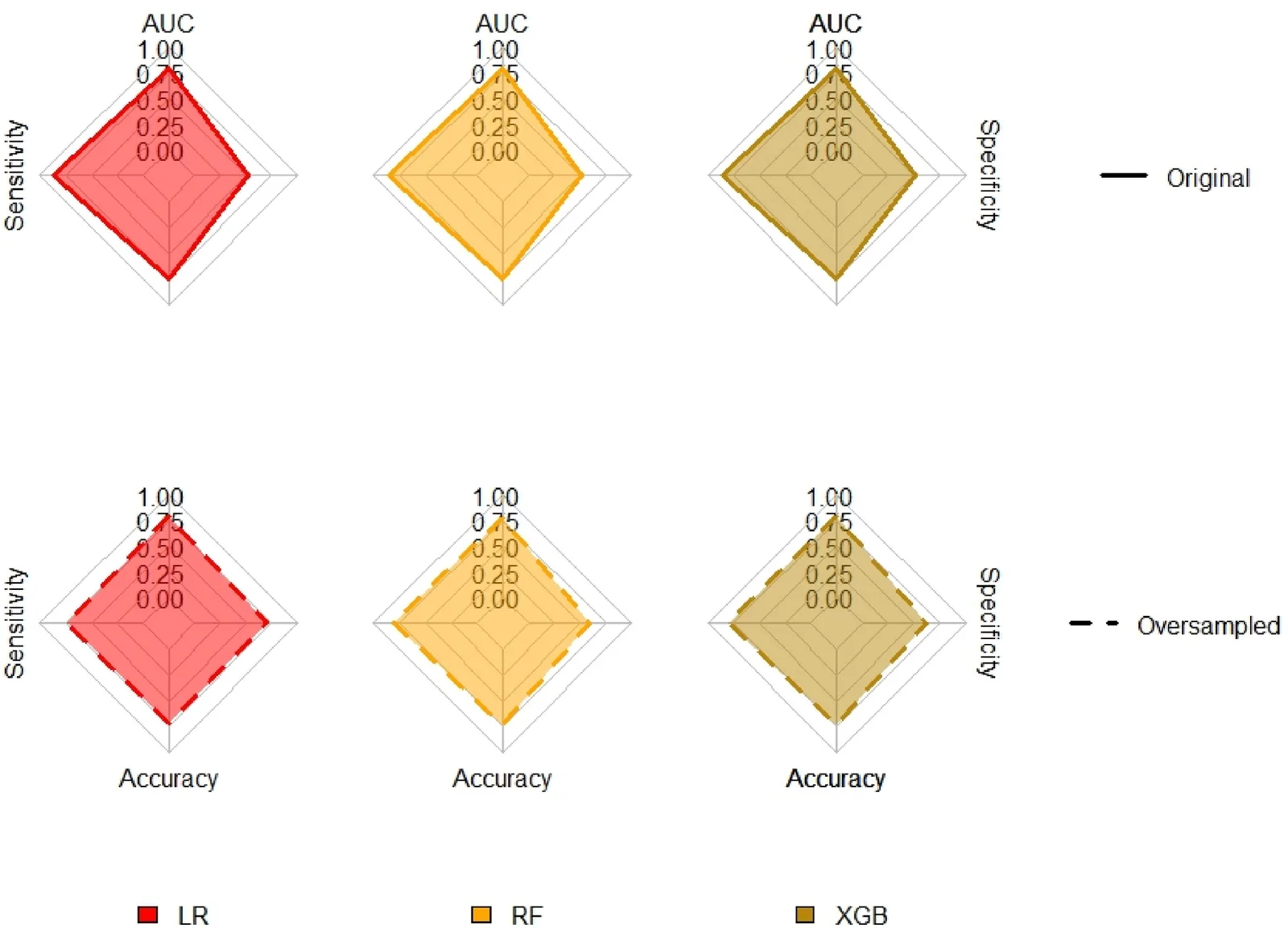
Performance metrics comparing original vs SMOTE data. The shaded areas indicate the levels of the performance metrics within each model/data combination.

We evaluated the performance of our models when trained on the original dataset versus the oversampled dataset. Specifically, we wanted to assess whether the oversampling technique, as described in Section 3.4, improved model performance overall and within specific subpopulations. Note that all models compared in this section used patient-level variables along with community-level SDOH variables as predictors. Table 2 presents the percentage changes in performance metrics after training the extreme gradient boosting model on the oversampled data compared to the original data. Overall, we saw a decrease in sensitivity by 2.73% and an overall increase in specificity of 6.20%. Almost every subpopulation also experienced a decrease in sensitivity and an increase in specificity. These changes were expected because the oversampling technique increases the number of instances in the minority class of the target variable, hence it balances the true positive and true negative rates. By losing some accuracy in true positive rates, the model gained some increase in the true negative rates. Applying the oversampling algorithm to the training dataset of the other three model types resulted in similar findings. The overall results and the trends in performance matrices are now shown in Fig. 2.

### 3.3. Variable importance

**Fig. 3.**
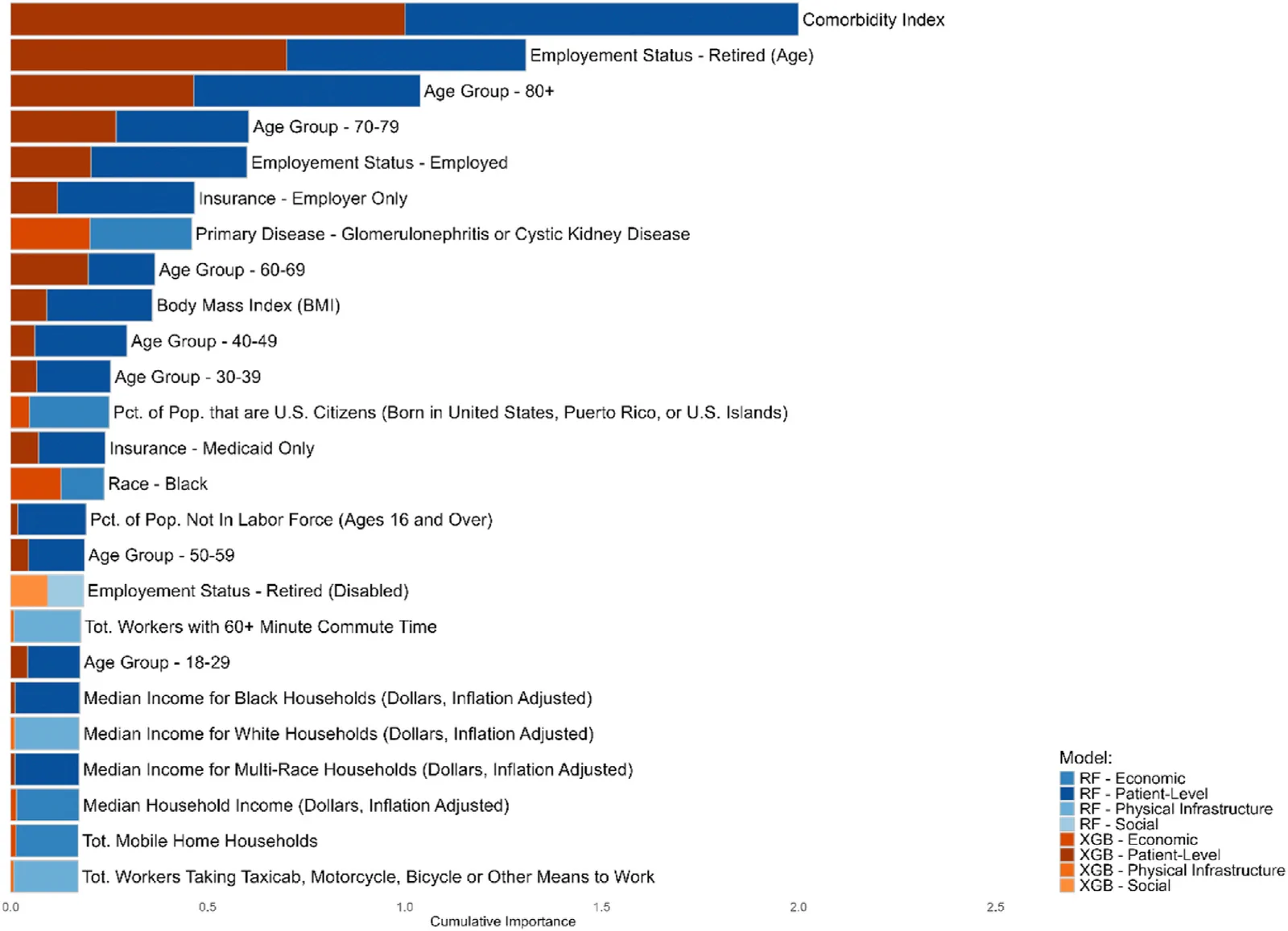
Top 25 variable importance values for RF and XGB models.

To assess the importance of both patient-level and community-level SDOH variables, we used variable importance values from the random forest and extreme gradient boosting models, along with significance levels from the logistic regression output. Fig. 3 displays the top 25 scaled importance values from the random forest and extreme gradient boosting models. Our analysis found that the most influential predictors of all-cause mortality in ESKD patients were patient-level factors, including comorbidity index, employment status, age group, primary disease, insurance type, and body mass index (BMI). Of the patient-level factors, employment status and insurance type are SDOH variables that are available at the patient level in the USRDS dataset. A challenge is they are not updated on a regular basis, only if there is a reportable event such as dialysis start, transplant, or death. However, it should be noted that the importance of the patient-level factor of these two variables was very strong when evaluating at both the patient-level in the USRDS dataset and the community-level in the AHRQ-SDOH dataset. Among the community-level SDOH variables, factors relating to citizenship, unemployment, and commute time also displayed some influence on mortality, though they were less impactful than the previously mentioned patient-level factors. Interestingly, several of the community-level SDOH variables achieve higher importance scores than the patient-level variables (like institutionalization or rurality) that do not appear in the top 25. Overall, the community-level SDOH variables appeared to be overpowered by the stronger influence of patient-level factors, limiting their additional contribution to the models.

The significance level of each variable in the logistic regression model is displayed in Table A.5. A low p-value provides evidence that the predictor variable is significantly associated with our target variable. In Table A.5, we observed that the same high importance patient-level variables from the tree ensemble models, along with several other patient-level variables, obtained the smallest level of significance, indicating their strong influence in predicting all-cause mortality in ESKD patients. The community-level SDOH variables with the smallest significance levels were Pct. of Pop. Consisting of U.S. Born Citizens, Tot. People Aged 0–17 in Poverty, and Pct. of Pop. Uninsured (138%–400% Poverty, Under 65 Years Old). Nearly all patient-level variables were found to be significant predictors of all-cause mortality in the logistic regression model, whereas the tree-based models identified fewer patient-level variables as being important, though these were highly predictive.

The variable importance results from the random forest and extreme gradient boosting models, alongside the significance levels from the logistic regression model, consistently highlighted the fact that patient-level factors, such as comorbidity index, employment status, age group, primary disease, insurance type, and BMI, were the most influential predictors of all-cause mortality in ESKD patients. While some community-level SDOH variables displayed mild importance, their impact on mortality prediction appeared to be limited when combined with patient-level variables, emphasizing the stronger influence of individual patient characteristics in these predictions.

### 3.4. Inference using logistic regression

All three ML models showed comparable performance metrics. The random forest and extreme gradient boosting, which can capture non-linearity in the data, performed similarly to the logistic regression model, a generalized linear model. Model calibration was evaluated by splitting the dataset into an 80% training and a 20% validation set to construct calibration curve and calculate Brier score. The calibration curve (Appendix Figure A.1) shows an agreement between predicted and observed risks, with calibration intercept of 0 (95% CI: −0.06, 0.03) and a slope of 0.99 (95% CI: 0.96–1.02). The overall predictive accuracy was supported by a Brier score of 0.173. Together, these metrics indicate that the model is well-calibrated and provides a reliable individualized risk prediction.

In addition, using the coefficient estimates from the logistic regression model, we examined the direction of the relationships between the predictor variables and the target variable. Table A.5. in the Appendix provides a summary of the logistic regression model output, including coefficient estimates. We find that generally as age group increased, the likelihood of death increased; as comorbidity index increased, the likelihood of death also increased; and for patients who were retired due to age, the likelihood of death increased. On the other hand, as BMI increased, the likelihood of death decreased. Additionally, for patients who were employed, the likelihood of death decreased. The finding that higher BMI was associated with lower mortality risk is consistent with the “obesity paradox” described by Park et al. [23]. They suggest that higher BMI, which reflects greater skeletal muscle mass and body fat, may be protective for patients undergoing hemodialysis.

Examining the community-level SDOH variables, some of the statistically significant associations with non-zero coefficients include, as the percentage of U.S. born citizens in a patient's ZIP code increased, the patient's likelihood of mortality increased; as the total number of hospices in a patient's county increased, the likelihood of death increased; and as the percent of uninsured individuals in a patient's county increases, the likelihood of death increases. On the other hand, as the total number of social associations in a patient's county increased, the patient's likelihood of death decreased.

## 4. Discussion

### Methodological findings:

Our methodological findings demonstrate that while ML methods such as random forest and extreme XGBoost can effectively predict all-cause mortality among patients with ESKD, patient-level variables remain the strongest contributors to predictive performance. However, when compared to the best-performing logistic regression and random forest, the performance metrics of the extreme gradient boosting model were not substantially better, but rather comparable to those of the other model types. This has been previously reported in other nephrology studies where ML models did not yield a large gain over traditional methods [24–26].

We also found that including community-level SDOH factors as predictor variables only led to a modest improvement in the overall predictive power of the logistic regression model. This limited impact may be attributed to the community-level nature of the SDOH variables used. Additionally, the rapid progression of ESKD may make patient-specific factors more influential, particularly in the later stages of the disease [27,28]. Furthermore, incorporating community-level SDOH variables into the models did not consistently lead to significant improvements in model performance across the different subpopulations [29,30].

The application of SMOTE did not enhance overall model performance or performance within subpopulations. Although SMOTE improved specificity, it led to a reduction in sensitivity, ultimately resulting in a decline in the models’ AUC. Reduced sensitivity carries the risk of false negatives, or missing opportunities for intensified monitoring people who are at higher risk but are missed by the model. This tradeoff is clinically significant as failing to identify high-risk patients could reduce opportunities for timely intervention.

### Clinical findings:

The clinical interpretation of our findings emphasizes how predictors manifest in patient outcomes and risk stratification in ESKD. Building on the results from the model performance analysis, our examination of variable importance and significance further emphasized the critical role of patient-level variables, particularly comorbidity index, employment status, age group, primary disease, insurance type, and BMI, as the most influential predictors of all-cause mortality in ESKD patients. However, our analysis of variable importance using the tree-based models revealed that certain community-level SDOH variables, such as citizenship rates, unemployment rates, and commute time, had a greater influence on mortality than some patient-level predictors. Given the comparable performance of the logistic regression model (a generalized linear model) and the random forest, extreme gradient boosting, and artificial neural network models, we used the coefficient estimates from the logistic regression model to assess the directionality of predictors in relation to mortality.

We found that patients with higher age, higher comorbidity index, and those retired due to age had a higher likelihood of death. In contrast, patients who were employed and patients with a higher BMI had a decreased likelihood of death. Regarding community-level SDOH variables, we observed that patients from communities with higher rates of uninsured residents had an increased likelihood of mortality while patients from communities with more social associations had a decreased likelihood of mortality. The statistically significant result of uninsured rates shows that community-level SDOH may still exert influence on mortality pathways in ESKD. These findings support previous evidence that both clinical and social environments jointly contribute to disparities in ESKD outcomes [30–32] and underscores the need for developing strategies to improve the disparities.

## 5. Limitations and future work

Several limitations should be acknowledged. First, our models rely on aggregated community-level SDOH variables, which may not fully capture the complexity of individual patient experiences across the disease trajectory. Similar limitations have been documented in prior studies using county- or ZIP Code level socio economic measures [31,32]. As SDOH data become increasingly available in medical records, future research could incorporate these patient-level measures to reflect individual social and environmental contexts more accurately into analysis and inference. As SDOH are recently being collected as EMR records, future research could incorporate this information to capture more accurate information at individual levels. In addition, the patient-level data in the USRDS dataset is not updated on a routine basis, only when there is a reportable event such as a modality change or death of the patient. Future research could also focus on extending this work to include longitudinal patient-level data, showing the disease progression until kidney failure particularly from electronic medical records (EMRs), which will allow for a deeper understanding of both disease progression and the role of social determinants of health to improve predictive accuracy. A primary methodological consideration of this study was the use of a binary mortality outcome rather than a time-to-event (survival-analysis). While survival models handle censoring more robustly, a binary framework was specifically selected to align with the available SDOH data. In clinical practice, identifying patients at high risk of mortality within a fixed (5-year) period is critical for long-term enrollment of social support and palliative care programs. Given that community-level SDOH variables are often static or sparsely updated, the binary approach allows for a direct assessment of the stable baseline risk contributed by a patient's environment and living condition at the time of ESKD onset. Future study could implement time-to-event survival ML models such as random survival forests (Survival-XGBoost) and deep learning-based (DeepSurv) survival models [33,34] to capture the time-to-event structure as well as the medical and non-medical factors that could be associated with mortality or disease progression for patients with kidney disease as health providers are adapting collection of SDOH data within EMR.

## 6. Conclusion

This study contributes to the growing body of work on ML-based mortality prediction in ESKD by integrating patient-level clinical predictors with community-level SDOH, while systematically evaluating oversampling methods. Our findings highlight the primacy of clinical factors in advanced disease, the limited role of community-level SDOH in late-stage prediction, and the mixed value of SMOTE for imbalanced clinical datasets. The value of our modeling efforts and the data used is essential groundwork that lays the foundation for future research. Future research would examine kidney disease across all stages with a focus on social determinants of health (SDOH), using both explicit and derived data from EMRs. The aim would be to collect and analyze this data longitudinally over time. One hypothesis is that SDOH may have a greater influence during the earlier stages of kidney disease. In addition to leveraging patient-level data, refinement in imbalance strategies and adopting survival-focused ML approaches to better capture the dynamic and multifactorial nature of ESKD outcomes is important. This next step is critical, as it can uncover patterns and disparities that are not apparent in higher-level analyses. If successful, this approach could significantly advance early detection strategies, inform targeted interventions, and contribute to better health outcomes for all.

## Supplementary Material

1

## Figures and Tables

**Table 1:** Model performance (mean AUC ± SD) across variable sets and models.

Model	SDOH	Patient Subset	Patient	Patient + SDOH

LR	0.575 (0.004)	0.784 (0.005)	0.791 (0.005)	0.792 (0.005)
RF	0.543 (0.006)	0.771 (0.006)	0.782 (0.006)	0.782 (0.007)
XGB	0.585 (0.003)	0.787 (0.006)	0.795 (0.005)	0.794 (0.006)

**Table 2:** Change in XGB subpopulation performance after SMOTE application.

Subpopulation	Accuracy % Change	Sensitivity % Change	Specificity % Change	AUC % Change

American Indian	−1.37%	−1.70%	−0.16%	−0.26%
Asian	−0.14%	−2.27%	2.54%	−0.42%
Black	−0.41%	−3.89%	5.66%	−0.17%
Native Hawaiian/Pacific Islander	0.02%	0.23%	−0.40%	−0.93%
Other	−2.41%	0.69%	−6.06%	0.04%
White	−0.09%	−1.97%	7.43%	−0.02%

Hispanic	0.33%	−2.71%	3.73%	−0.01%
Non-Hispanic	−0.27%	−2.33%	7.04%	−0.15%

Isolated Small Rural	−0.08%	−1.73%	8.66%	−0.06%
Small Rural	0.07%	−1.25%	6.12%	−0.10%
Large Rural	−0.35%	−1.77%	6.05%	−0.15%
Urban	−0.19%	−2.52%	6.16%	−0.14%

Midwest	−0.13%	−2.06%	7.19%	−0.07%
Northeast	−0.17%	−1.83%	5.35%	−0.21%
South	−0.20%	−2.51%	6.80%	−0.14%
West	−0.22%	−2.89%	5.18%	−0.14%

Overall	−0.19%	−2.37%	6.20%	−0.14%

## Appendix A. Supplementary data

Supplementary data to this article can be found online at https://doi.org/10.1016/j.imu.2026.101779.
