# Supplementary material for: The prediction of all-cause mortality in end-stage kidney disease patients using social determinants of health: A machine learning framework
Source: Inform Med Unlocked. Author manuscript; Available in PMC 2026 Sep 16. (PMC13577197; doi:10.1016/j.imu.2026.101779)
Supplement: Supplementary file 1 [file NIHMS2200339-supplement-1.docx]

## Appendix A. Supplemental Tables

Table A.1 contains a summary of the USRDS and AHRQ-SDOH variables used for final selection, including the variable names, definitions, and level at which data was evaluated. Table A.2 contains the baseline characteristics of the USRDS data. Table A.3. has the baseline characteristics comparing the complete-case data with missingness. Table A.4. shows model performance for all the considered models. Table A.5. shows the full logistic regression model fit. Finally, Figure A.1 shows the Calibration curve with corresponding statistics of the logistic regression model.

Table A.1. USRDS and AHRQ-SDOH Variables Used in Machine Learning Model Analysis

| **Variable Name** | **Dataset** | **Data Level** | **Definition** |
| --- | --- | --- | --- |
| Event | USRDS | Patient | All-cause mortality by the end of the follow- up period; one indicates the patient experienced death, zero indicates the patient did not experience death |
| Age | USRDS | Patient | Age of patient, recorded in years |
| Age Group | USRDS | Patient | Grouped version of Age variable: Younger than 18, 18-29, 30-39, 40-49, 50-59, 60-69, 70-79, 80 and older |
| Sex | USRDS | Patient | Sex of patient: Male, Female |
| Race | USRDS | Patient | Race of patient: White, Black, Asian, American Indian, Native Hawaiian/Pacific Islander, Other |
| Hispanic | USRDS | Patient | Indicates if patient selected Hispanic on registration form: Yes, No |
| Primary Disease | USRDS | Patient | Primary disease indicated on patient registration form: Diabetes, Hypertension, Glomerulonephritis, or Cystic Kidney Disease, Other |
| Rurality | USRDS | Patient | Rurality of patient residence: Urban, Large Rural, Small Rural, Isolated Small Rural |
| Comorbidity Index | USRDS | Patient | Index summarizing the comorbidities indicated by patient on registration form |
| Inability to Ambulate | USRDS | Patient | Inability to ambulate indicated by patient on registration form: No, Yes |
| Inability to Transfer | USRDS | Patient | Inability to transfer indicated by patient on registration form: No, Yes |
| Institutionalized | USRDS | Patient | Institution indicated by patient on registration form, if any: No, Nursing Home, Assisted Living, Other Institution |
| Needs Assistance with Daily Activities | USRDS | Patient | Needs assistance with daily activities indicated by patient on registration form: No, Yes |
| Alcohol Dependence | USRDS | Patient | Alcohol dependence indicated by patient on registration form: No, Yes |
| Tobacco Use | USRDS | Patient | Tobacco use indicated by patient on registration form: No, Yes |
| Drug Dependence | USRDS | Patient | Drug dependence indicated by patient on registration form: No, Yes |
| Amputation | USRDS | Patient | Amputation indicated by patient on registration form: No, Yes |
| Toxic Nephropathy | USRDS | Patient | Toxic nephropathy indicated by patient on registration form: No, Yes |
| Modality | USRDS | Patient | Patient modality (treatment method): Hemodialysis, Peritoneal Dialysis |
| Employment | USRDS | Patient | Patient employment status: Unemployed, Employed, Retired-Age, Retired-Disabled, Other |
| Insurance | USRDS | Patient | Insurance type of patient: Employer Only, Medicaid only, Medicare Only, DNV+, Medicare and Employer, Medicare and Medicaid, Multiple Otherwise, Other, None |
| BMI | USRDS | Patient | Patient body mass index calculated from height and weight provided on registration form |
| Region | USRDS | Patient | Region of patient residence: West, South, Midwest, Northeast |
| Zip Code | USRDS | Patient | ZIP code of patient residence |
| County | USRDS | Patient | County FIPS code of patient residence |
| State | USRDS | Patient | State of patient residence |
| State FIPS | USRDS | Patient | State FIPS code of patient residence |
| Year | USRDS | Patient | Year patient filled out registration form |
| Pct. of Pop. Uninsured (138%-400% Poverty, Under 65 Years Old, Estimate) | AHRQ-SDOH | County | Estimated percentage of uninsured population between 138%-400% of poverty (under 65 years) |
| Pct. of Pop. that are U.S. Citizens (Born in United States, Puerto Rico, or U.S. Islands) | AHRQ-SDOH | ZIP Code | Percentage of population consisting of U.S. citizens born in United States, Puerto Rico, or U.S. Islands (ZCTA level) |
| Tot. Pop. in Poverty (Aged 0-17, Estimate) | AHRQ-SDOH | County | Estimated number of people aged 0-17 in poverty |
| Tot. Hospices (Per 1000 Pop.) | AHRQ-SDOH | County | Total number of hospices per 1,000 population |
| Tot. Households with Monthly Owner Costs of At Least 30 Pct. Household Income | AHRQ-SDOH | ZIP Code | Total number of owner-occupied housing units with selected monthly owner costs equal to 30 percent or more of household income (ZCTA level) |
| Tot. Social Associations | AHRQ-SDOH | ZIP Code | Total number of social associations |
| Tot. Nurse Practitioners with NPI (Per 1000 Pop.) | AHRQ-SDOH | County | Total number of nurse practitioners with NPI per 1,000 population |
| Tot. Pop. with a Bachelor's Degree (Ages 25 and Over) | AHRQ-SDOH | ZIP Code | Total number of population with a bachelor's degree (ages 25 and over, ZCTA level) |
| Tot. Mobile Home Households | AHRQ-SDOH | ZIP Code | Total number of housing units that are mobile homes (ZCTA level) |
| Pct. of Adults Who Are Current Smokers | AHRQ-SDOH | County | Percentage of adults who are current smokers |
| Tot. Pop. with a High School Diploma (Ages 25 and Over) | AHRQ-SDOH | ZIP Code | Total number of population with only high school diploma (ages 25 and over, ZCTA level) |
| Tot. Family Households with No Spouse Present | AHRQ-SDOH | ZIP Code | Total number of family households with female or male householder, no spouse present (ZCTA level) |
| Pct. of Medicare Beneficiaries with Drug Plan (Per 100 People) | AHRQ-SDOH | County | Percentage of Medicare eligible beneficiaries enrolled in a prescription drug plan per 100 population |
| Tot. Non-Federal General Preventive Medicine Specialists | AHRQ-SDOH | County | Total number of non-federal general preventive medicine specialists |
| Median Income for Asian Households (Dollars, Inflation Adjusted) | AHRQ-SDOH | ZIP Code | Median household income for households with an Asian alone householder (dollars, inflation-adjusted to data file year, ZCTA level) |
| Tot. Food Distribution Program on Indian Reservations (FDPIR) Sites | AHRQ-SDOH | County | Total number of the Food Distribution Program on Indian Reservations (FDPIR) sites |
| Median Household Income (Dollars, Inflation Adjusted) | AHRQ-SDOH | ZIP Code | Median household income (dollars, inflation-adjusted to data file year, ZCTA level) |
| Pct. of Pop. Not In Labor Force (Ages 16 and Over) | AHRQ-SDOH | ZIP Code | Percentage of population not in labor force (ages 16 and over, ZCTA level) |
| Median Income for American Indian or Alaskan Native Households (Dollars, Inflation Adjusted) | AHRQ-SDOH | ZIP Code | Median household income for households with an American Indian and Alaska Native alone householder (dollars, inflation-adjusted to data file year, ZCTA level) |
| Median Income for Hispanic or Latino Households (Dollars) | AHRQ-SDOH | County | Median household income for households with a Hispanic or Latino householder (dollars, inflation-adjusted to data file year) |
| Pct. Medicaid Eligible Beneficiaries Enrolled in a Medicare Advantage Plan (Per 100 Pop.) | AHRQ-SDOH | County | Percentage of Medicare eligible beneficiaries enrolled in a Medicare Advantage plan per 100 population |
| Tot. Pop. with Public-Only Health Insurance | AHRQ-SDOH | ZIP Code | Total number of population with other public-only health insurance combinations (ZCTA level) |
| Median Income for White Households (Dollars, Inflation Adjusted) | AHRQ-SDOH | ZIP Code | Median household income for households with a White alone, not Hispanic or Latino householder (dollars, inflation-adjusted to data file year, ZCTA level) |
| Tot. Beds in Long-Term Hospitals (Per 1000 Pop.) | AHRQ-SDOH | County | Total number of beds in long-term hospital per 1,000 population |
| Tot. Pop. with Master's or Professional School Degree or Doctorate (Ages 25 and Over) | AHRQ-SDOH | ZIP Code | Total number of population with a master's or professional school degree or doctorate (ages 25 and over, ZCTA level) |
| Median Income for Multi-Race Households (Dollars, Inflation Adjusted) | AHRQ-SDOH | ZIP Code | Median household income for households with a two or more races householder (dollars, inflation-adjusted to data file year, ZCTA level) |
| Gini Index of Income Inequality | AHRQ-SDOH | ZIP Code | Gini index of income inequality (ZCTA level) |
| Tot. Employed in Education Services, Healthcare, and Social Assistance (Ages 16 and Over) | AHRQ-SDOH | ZIP Code | Total number of employed working in educational services, and healthcare and social assistance (ages 16 and over, ZCTA level) |
| Tot. Pop. with Employer-Based and Medicare Health Insurance | AHRQ-SDOH | ZIP Code | Total number of population with employer-based and Medicare insurance coverage (ZCTA level) |
| Pct. Medicaid Eligible Medicare FFS Beneficiaries (Eligible For At Least One Month) | AHRQ-SDOH | County | Percentage of Medicare FFS beneficiaries who are eligible for Medicaid for at least one month |
| Tot. Non-Federal MDs | AHRQ-SDOH | County | Total number of non-federal MDs |
| Tot. Pop. with Employer-Based and Direct Purchase Health Insurance | AHRQ-SDOH | ZIP Code | Total number of population with employer-based and direct-purchase coverage (ZCTA level) |
| Tot. Pop. with Other Health Insurance | AHRQ-SDOH | ZIP Code | Total number of population with other health insurance coverage combinations (ZCTA level) |
| Tot. Pop. With Income to Poverty Ratio Under 1.00 | AHRQ-SDOH | ZIP Code | Total number of population with an income to poverty ratio under 1.00 (ZCTA level) |
| Tot. Households with a Person Living Alone (Ages 65 and Over) | AHRQ-SDOH | ZIP Code | Total number of households with a person 65 years and over living alone (ZCTA level) |
| Tot. Workers Taking Taxicab, Motorcycle, Bicycle or Other Means to Work | AHRQ-SDOH | ZIP Code | Total number of workers taking taxicab, motorcycle, bicycle, or other means to work (ages 16 and over, ZCTA level) |
| Tot. Pop. with Household Income over $100,000 | AHRQ-SDOH | ZIP Code | Total number of population with household income greater than $100,000 (ZCTA level) |
| Tot. Workers Taking Public Transportation (>15 Minute Commute Time) | AHRQ-SDOH | ZIP Code | Total number of workers who use public transportation with < 15-minute commute time (ages 16 and over, ZCTA level) |
| Tot. Short-Term General Hospitals (Per 1000 Pop.) | AHRQ-SDOH | County | Total number of short-term general hospitals per 1,000 population |
| Median Income for Other Race Households (Dollars, Inflation Adjusted) | AHRQ-SDOH | ZIP Code | Median household income for households with a some other race alone householder (dollars, inflation-adjusted to data file year, ZCTA level) |
| Tot. Civilian Veterans with some College Education or Associate's Degree (Ages 25 and Over) | AHRQ-SDOH | ZIP Code | Total number of civilian veterans that have some college education or an associate's degree (ages 25 and over, ZCTA level) |
| Tot. Pop. with Medicare Only | AHRQ-SDOH | ZIP Code | Total number of population with Medicare only (ZCTA level) |
| Tot. Households with Monthly Owner Costs of At Least 50 Pct. Household Income | AHRQ-SDOH | ZIP Code | Total number of occupied housing units: rented (ZCTA level) |
| Min Distance to Nearest Medical-Surgical ICU (Miles) | AHRQ-SDOH | County | Minimum distance in miles to the nearest medical-surgical ICU, calculated using population weighted tract centroids in the county |
| Median Income for Black Households (Dollars, Inflation Adjusted) | AHRQ-SDOH | ZIP Code | Median household income for households with a Black or African American alone householder (dollars, inflation-adjusted to data file year, ZCTA level) |
| Tot. Supermarkets and Other Grocery (Except Convenience) Stores (Per 1000 Pop.) | AHRQ-SDOH | County | Total number of supermarkets and other grocery (except convenience) stores per 1,000 people |
| Max Distance to Nearest Urgent Care (Miles) | AHRQ-SDOH | County | Maximum distance in miles to the nearest urgent care, calculated using population weighted tract centroids in the county |
| Min Distance to Nearest Urgent Care (Miles) | AHRQ-SDOH | County | Minimum distance in miles to the nearest urgent care, calculated using population weighted tract centroids in the county |
| Tot. Physician Assistants with NPI (Per 1000 Pop.) | AHRQ-SDOH | County | Total number of physician assistants with NPI per 1,000 population |
| Tot. Employed in Agriculture, Forestry, Fishing, Hunting, and Mining (Ages 16 and Over) | AHRQ-SDOH | ZIP Code | Total number of employed working in agriculture, forestry, fishing and hunting, and mining (ages 16 and over, ZCTA level) |
| Tot. Pop. with Income to Poverty Ratio Under 0.50 | AHRQ-SDOH | ZIP Code | Total number of population with income to poverty ratio under 0.50 (ZCTA level) |
| Max Distance to Nearest Health Clinic (FQHC, RHC) (Miles) | AHRQ-SDOH | County | Maximum distance in miles to the nearest health clinic (FQHC, RHC), calculated using population weighted tract centroids in the county |
| Tot. Civilian Veterans Who Are At Most High School Graduates (Ages 25 and Over) | AHRQ-SDOH | ZIP Code | Total number of civilian veterans who are high school graduates or less than high school graduates (age 25 years and over, ZCTA level) |
| Tot. Pop. with Employer-Based Health Insurance | AHRQ-SDOH | ZIP Code | Total number of population with employer-based health insurance (ZCTA level) |
| Tot. Medical Specialists | AHRQ-SDOH | County | Total number of medical specialists |
| Pct. Pop. Lacking Adequate Food Access | AHRQ-SDOH | County | Percentage of population who lack adequate access to food |
| Median Income for Native Hawaiian and Pacific Islander Households (Dollars, Inflation Adjusted) | AHRQ-SDOH | ZIP Code | Median household income for households with a Native Hawaiian and other Pacific Islander alone householder (dollars, inflation-adjusted to data file year, ZCTA level) |
| Pct. of Pop. in Poverty (Aged 0-17, Estimate) | AHRQ-SDOH | County | Estimated percentage of people aged 0-17 in poverty |
| Tot. Workers with 60+ Minute Commute Time | AHRQ-SDOH | ZIP Code | Total number of workers with at least 60-minute commute time (ages 16 and over, ZCTA level) |

Table A.2. Baseline Characteristics by Event Status

| **Characteristic** | **Overall**  N = 108,975^1^ | **0**  N = 39,049 (35.8%)^1^ | **1**  N = 69,926 (64.2%)^1^ | **p-value**^2^ |
| --- | --- | --- | --- | --- |
| **Age, mean (SD)** | 62.8 (15.2) | 54.5 (15.1) | 67.4 (13.2) | <0.001 |
| **Age group** |  |  |  | <0.001 |
| 18-29 | 2,235 (2.1%) | 1,798 (4.6%) | 437 (0.6%) |  |
| 30-39 | 5,047 (4.6%) | 3,546 (9.1%) | 1,501 (2.1%) |  |
| 40-49 | 10,424 (9.6%) | 6,438 (16%) | 3,986 (5.7%) |  |
| 50-59 | 20,015 (18%) | 9,928 (25%) | 10,087 (14%) |  |
| 60-69 | 29,086 (27%) | 10,186 (26%) | 18,900 (27%) |  |
| 70-79 | 25,191 (23%) | 5,092 (13%) | 20,099 (29%) |  |
| 80 and older | 16,410 (15%) | 1,558 (4.0%) | 14,852 (21%) |  |
| Younger than 18 | 567 (0.5%) | 503 (1.3%) | 64 (<0.1%) |  |
| **Sex** |  |  |  | 0.002 |
| Female | 46,127 (42%) | 16,289 (42%) | 29,838 (43%) |  |
| Male | 62,848 (58%) | 22,760 (58%) | 40,088 (57%) |  |
| **Race** |  |  |  | <0.001 |
| American Indian | 972 (0.9%) | 392 (1.0%) | 580 (0.8%) |  |
| Asian | 4,284 (3.9%) | 2,082 (5.3%) | 2,202 (3.1%) |  |
| Black | 29,016 (27%) | 12,678 (32%) | 16,338 (23%) |  |
| Native Hawaiian / Pacific Islander | 1,160 (1.1%) | 612 (1.6%) | 548 (0.8%) |  |
| Other | 312 (0.3%) | 158 (0.4%) | 154 (0.2%) |  |
| White | 73,231 (67%) | 23,127 (59%) | 50,104 (72%) |  |
| **Hispanic ethnicity** | 16,154 (15%) | 7,891 (20%) | 8,263 (12%) | <0.001 |
| **Primary disease** |  |  |  | <0.001 |
| Diabetes | 52,016 (48%) | 16,447 (42%) | 35,569 (51%) |  |
| Glomerulonephritis or Cystic kidney disease | 10,201 (9.4%) | 6,215 (16%) | 3,986 (5.7%) |  |
| Hypertension | 32,515 (30%) | 11,658 (30%) | 20,857 (30%) |  |
| Other | 14,243 (13%) | 4,729 (12%) | 9,514 (14%) |  |
| **Comorbidity index, mean (SD)** | 2.7 (2.6) | 1.7 (2.0) | 3.2 (2.6) | <0.001 |
| **BMI, mean (SD)** | 29.6 (8.0) | 30.1 (8.1) | 29.4 (8.0) | <0.001 |
| **Inability to ambulate** | 7,621 (7.0%) | 1,089 (2.8%) | 6,532 (9.3%) | <0.001 |
| **Inability to transfer** | 4,099 (3.8%) | 534 (1.4%) | 3,565 (5.1%) | <0.001 |
| **Needs assistance with daily activities** | 14,501 (13%) | 2,684 (6.9%) | 11,817 (17%) | <0.001 |
| **Institutionalized** |  |  |  | <0.001 |
| Assisted living | 704 (0.6%) | 104 (0.3%) | 600 (0.9%) |  |
| No | 99,638 (91%) | 37,786 (97%) | 61,852 (88%) |  |
| Nursing home | 7,951 (7.3%) | 998 (2.6%) | 6,953 (9.9%) |  |
| Other institution | 682 (0.6%) | 161 (0.4%) | 521 (0.7%) |  |
| **Alcohol dependence** | 1,678 (1.5%) | 561 (1.4%) | 1,117 (1.6%) | 0.041 |
| **Tobacco use** | 6,451 (5.9%) | 1,997 (5.1%) | 4,454 (6.4%) | <0.001 |
| **Drug dependence** | 1,281 (1.2%) | 495 (1.3%) | 786 (1.1%) | 0.038 |
| **Amputation** | 3,481 (3.2%) | 785 (2.0%) | 2,696 (3.9%) | <0.001 |
| **Toxic nephropathy** | 432 (0.4%) | 154 (0.4%) | 278 (0.4%) | >0.9 |
| **Modality** |  |  |  | <0.001 |
| Hemodialysis | 99,960 (92%) | 34,353 (88%) | 65,607 (94%) |  |
| Peritoneal dialysis | 9,015 (8.3%) | 4,696 (12%) | 4,319 (6.2%) |  |
| **Employment** |  |  |  | <0.001 |
| Employed | 11,783 (11%) | 7,911 (20%) | 3,872 (5.5%) |  |
| Other | 3,262 (3.0%) | 2,126 (5.4%) | 1,136 (1.6%) |  |
| Retired-Age | 42,053 (39%) | 8,185 (21%) | 33,868 (48%) |  |
| Retired-Disabled | 21,490 (20%) | 6,962 (18%) | 14,528 (21%) |  |
| Unemployed | 30,387 (28%) | 13,865 (36%) | 16,522 (24%) |  |
| **Insurance** |  |  |  | <0.001 |
| DVA+ | 2,014 (1.8%) | 568 (1.5%) | 1,446 (2.1%) |  |
| Employer only | 12,965 (12%) | 8,244 (21%) | 4,721 (6.8%) |  |
| Medicaid only | 13,911 (13%) | 7,409 (19%) | 6,502 (9.3%) |  |
| Medicare and Employer | 5,948 (5.5%) | 1,749 (4.5%) | 4,199 (6.0%) |  |
| Medicare and Medicaid | 14,510 (13%) | 3,747 (9.6%) | 10,763 (15%) |  |
| Medicare only | 33,877 (31%) | 7,674 (20%) | 26,203 (37%) |  |
| Multiple otherwise | 14,333 (13%) | 3,217 (8.2%) | 11,116 (16%) |  |
| None | 5,069 (4.7%) | 3,063 (7.8%) | 2,006 (2.9%) |  |
| Other | 6,348 (5.8%) | 3,378 (8.7%) | 2,970 (4.2%) |  |
| **Rurality** |  |  |  | <0.001 |
| Isolated small rural | 2,765 (2.5%) | 811 (2.1%) | 1,954 (2.8%) |  |
| Large rural | 9,806 (9.0%) | 2,997 (7.7%) | 6,809 (9.7%) |  |
| Small rural | 4,752 (4.4%) | 1,481 (3.8%) | 3,271 (4.7%) |  |
| Urban | 91,652 (84%) | 33,760 (86%) | 57,892 (83%) |  |
| **Region** |  |  |  | <0.001 |
| Midwest | 21,852 (20%) | 7,177 (18%) | 14,675 (21%) |  |
| Northeast | 18,041 (17%) | 6,126 (16%) | 11,915 (17%) |  |
| South | 46,061 (42%) | 16,369 (42%) | 29,692 (42%) |  |
| West | 23,021 (21%) | 9,377 (24%) | 13,644 (20%) |  |
| ^1^Mean (SD); n (%) | | | | |
| ^2^Wilcoxon rank sum test; Pearson's Chi-squared test | | | | |

Table A.3. Comparison of baseline characteristics of the complete-case cohort and the data with missingness.

| **Category** | **Subgroup** | **DF1** | **DF2** | **Difference** |
| --- | --- | --- | --- | --- |
| Primary Condition | Diabetes | 0.477 (0.0015) | 0.462 (0.0036) | -0.016 |
|  | Hypertension | 0.298 (0.0014) | 0.286 (0.0033) | -0.012 |
|  | Glomerulonephritis / Cystic kidney disease | 0.094 (0.0009) | 0.099 (0.0022) | 0.006 |
|  | Other | 0.131 (0.0010) | 0.130 (0.0025) | -0.001 |
| Race | White | 0.672 (0.0014) | 0.675 (0.0034) | 0.004 |
|  | Black | 0.266 (0.0013) | 0.259 (0.0032) | -0.007 |
|  | Asian | 0.039 (0.0006) | 0.040 (0.0014) | 0.001 |
|  | American Indian | 0.009 (0.0003) | 0.009 (0.0007) | -0.001 |
|  | Native Hawaiian / Pacific Islander | 0.011 (0.0003) | 0.012 (0.0008) | 0.002 |
|  | Other | 0.003 (0.0002) | 0.003 (0.0004) | 0.000 |
| Sex | Male | 0.577 (0.0015) | 0.580 (0.0036) | 0.003 |
|  | Female | 0.423 (0.0015) | 0.420 (0.0036) | -0.003 |
| Age | Younger than 18 | 0.005 (0.0002) | 0.007 (0.0006) | 0.002 |
|  | 18-29 | 0.020 (0.0004) | 0.021 (0.0011) | 0.001 |
|  | 30-39 | 0.046 (0.0006) | 0.048 (0.0016) | 0.001 |
|  | 40-49 | 0.096 (0.0009) | 0.098 (0.0022) | 0.003 |
|  | 50-59 | 0.184 (0.0012) | 0.187 (0.0029) | 0.003 |
|  | 60-69 | 0.267 (0.0013) | 0.268 (0.0032) | 0.001 |
|  | 70-79 | 0.231 (0.0013) | 0.226 (0.0031) | -0.005 |
|  | 80 and older | 0.151 (0.0011) | 0.145 (0.0026) | -0.006 |

Table A.4. Full model performance metrics (mean ± SD)

| **Model** | **Variable Set** | **Accuracy** | **Sensitivity** | **Specificity** | **AUC** |
| --- | --- | --- | --- | --- | --- |
| LR | SDOH | 0.6411 (0.0048) | 0.9860 (0.0013) | 0.0235 (0.0031) | 0.5749  (0.0040) |
| LR | Patient subset | 0.7356 (0.0045) | 0.8554 (0.0050) | 0.5212 (0.0051) | 0.7840  (0.0053) |
| LR | Patient | 0.7392 (0.0044) | 0.8565 (0.0043) | 0.5291 (0.0055) | 0.7914  (0.0050) |
| LR | Patient & SDOH | 0.7394 (0.0043) | 0.8560 (0.0044) | 0.5305 (0.0053) | **0.7923 (0.0054)** |
| RF | SDOH | 0.6075 (0.0053) | 0.8145 (0.0082) | 0.2366 (0.0149) | 0.5432  (0.0062) |
| RF | Patient subset | 0.7341 (0.0041) | 0.8531 (0.0055) | 0.5210 (0.0109) | 0.7712  (0.0062) |
| RF | Patient | 0.7383 (0.0042) | 0.8548 (0.0044) | 0.5296 (0.0123) | **0.7823 (0.0057)** |
| RF | Patient & SDOH | 0.7360 (0.0047) | 0.8646 (0.0054) | 0.5056 (0.0142) | 0.7820  (0.0070) |
| XGB | SDOH | 0.6419 (0.0050) | 0.9871 (0.0014) | 0.0238 (0.0034) | 0.5848  (0.0033) |
| XGB | Patient subset | 0.7375 (0.0048) | 0.8627 (0.0050) | 0.5133 (0.0066) | 0.7865  (0.0056) |
| XGB | Patient | 0.7424 (0.0036) | 0.8623 (0.0030) | 0.5276 (0.0055) | **0.7947 (0.0053)** |
| XGB | Patient & SDOH | 0.7423 (0.0037) | 0.8624 (0.0045) | 0.5271 (0.0083) | 0.7942  (0.0056) |

Table A.5.The summary output of the logistic regression model, including variable names, coefficient estimates, standard errors, and p-values. Values are rounded to 4 decimal places, and for parameter estimates and standard errors, ~0.0000 or ~-0.0000 is used if rounded caused the value to be zero.

| **Variable** | **Estimate** | | **Std. Error** | **p-value** |
| --- | --- | --- | --- | --- |
| (Intercept) | | -1.4020 | 0.2409 | <0.0000 |
| Age Group - 18-29 | | 0.6071 | 0.1482 | <0.0000 |
| Age Group - 30-39 | | 0.9405 | 0.1417 | <0.0000 |
| Age Group - 40-49 | | 1.1800 | 0.1398 | <0.0000 |
| Age Group - 50-59 | | 1.4530 | 0.1391 | <0.0000 |
| Age Group - 60-69 | | 1.8020 | 0.1390 | <0.0000 |
| Age Group - 70-79 | | 2.3400 | 0.1401 | <0.0000 |
| Age Group - 80+ | | 3.1830 | 0.1420 | <0.0000 |
| Sex | | 0.0199 | 0.0152 | 0.1888 |
| Hispanic | | -0.6152 | 0.0246 | <0.0000 |
| Comorbidity Index | | 0.1451 | 0.0036 | <0.0000 |
| Inability to Ambulate | | 0.2770 | 0.0478 | <0.0000 |
| Inability to Transfer | | 0.1247 | 0.0642 | 0.0519 |
| Needs Assistance with Daily Activities | | 0.1889 | 0.0289 | <0.0000 |
| Alcohol Dependence | | 0.2691 | 0.0590 | <0.0000 |
| Tobacco Use | | 0.3179 | 0.0320 | <0.0000 |
| Drug Dependence | | 0.3528 | 0.0652 | <0.0000 |
| Amputation | | 0.3297 | 0.0457 | <0.0000 |
| Toxic Nephropathy | | 0.0826 | 0.1147 | 0.4714 |
| Modality | | -0.1898 | 0.0264 | <0.0000 |
| Race - Black | | -0.5332 | 0.0199 | <0.0000 |
| Race - Asian | | -0.7207 | 0.0395 | <0.0000 |
| Race - Native Hawaiian / Pacific Islander | | -0.6022 | 0.0687 | <0.0000 |
| Race - American Indian / Alaskan Native | | -0.3701 | 0.0775 | <0.0000 |
| Race - Other | | -0.5983 | 0.1310 | <0.0000 |
| Primary Disease - Glomerulonephritis or Cystic Kidney Disease | | -0.7224 | 0.0271 | <0.0000 |
| Primary Disease - Hypertension | | -0.2373 | 0.0179 | <0.0000 |
| Primary Disease - Other | | -0.1250 | 0.0242 | <0.0000 |
| Institutionalized - Nursing Home | | 0.4470 | 0.0412 | <0.0000 |
| Institutionalized - Assisted Living | | 0.2995 | 0.1145 | 0.0089 |
| Institutionalized - Other | | 0.1350 | 0.1036 | 0.1929 |
| Employment Status - Employed | | -0.4948 | 0.0284 | <0.0000 |
| Employment Status - Retired (Age) | | 0.0298 | 0.0234 | 0.2038 |
| Employment Status - Retired (Disabled) | | 0.1005 | 0.0215 | <0.0000 |
| Employment Status - Other | | -0.2631 | 0.0449 | <0.0000 |
| Insurance - Multiple Excluding Other Categories | | -0.0607 | 0.0263 | 0.021 |
| Insurance - Only Both Medicare and Medicaid | | 0.1116 | 0.0256 | <0.0000 |
| Insurance - Medicaid Only | | -0.3132 | 0.0266 | <0.0000 |
| Insurance - Employer Only | | -0.4932 | 0.0292 | <0.0000 |
| Insurance - Only Both Medicare and Employer | | -0.1405 | 0.0347 | 0.0001 |
| Insurance - Other | | -0.4084 | 0.0326 | <0.0000 |
| Insurance - None | | -0.4986 | 0.0367 | <0.0000 |
| Insurance - DVA Alone or with any Other Insurance | | -0.1500 | 0.0558 | 0.0072 |
| Rurality - Large Rural | | 0.0225 | 0.0307 | 0.4634 |
| Rurality - Small Rural | | -0.0853 | 0.0430 | 0.0474 |
| Rurality - Isolated Small Rural | | -0.0515 | 0.0557 | 0.3551 |
| Body Mass Index (BMI) | | -0.0100 | 0.0010 | <0.0000 |
| Pct. of Pop. that are U.S. Citizens (Born in United States, Puerto Rico, or U.S. Islands) | | 0.0041 | 0.0011 | 0.0001 |
| Tot. Family Households with No Spouse Present | | 0.0000 | 0.0000 | 0.0800 |
| Tot. Employed in Education Services, Healthcare, and Social Assistance (Ages 16 and Over) | | 0.0000 | 0.0000 | 0.3244 |
| Tot. Employed in Agriculture, Forestry, Fishing, Hunting, and Mining (Ages 16 and Over) | | 0.0000 | 0.0000 | 0.7907 |
| Pct. of Pop. Not In Labor Force (Ages 16 and Over) | | 0.0023 | 0.0015 | 0.1223 |
| Gini Index of Income Inequality | | 0.2324 | 0.2268 | 0.3055 |
| Median Income for American Indian or Alaskan Native Households (Dollars, Inflation Adjusted) | | ~0.0000 | ~0.0000 | 0.1415 |
| Median Income for Asian Households (Dollars, Inflation Adjusted) | | ~ -0.0000 | ~0.0000 | 0.1062 |
| Median Income for Black Households (Dollars, Inflation Adjusted) | | ~ -0.0000 | ~0.0000 | 0.6707 |
| Median Income for Multi-Race Households (Dollars, Inflation Adjusted) | | ~ -0.0000 | ~0.0000 | 0.2847 |
| Median Income for Native Hawaiian and Pacific Islander Households (Dollars, Inflation Adjusted) | | ~0.0000 | ~0.0000 | 0.9550 |
| Median Income for Other Race Households (Dollars, Inflation Adjusted) | | ~0.0000 | ~0.0000 | 0.5891 |
| Median Income for White Households (Dollars, Inflation Adjusted) | | ~0.0000 | ~0.0000 | 0.2641 |
| Median Household Income (Dollars, Inflation Adjusted) | | ~ -0.0000 | ~ 0.0000 | 0.1123 |
| Tot. Pop. with Household Income over $100,000 | | 0.0000 | 0.0000 | 0.4236 |
| Tot. Pop. with Income to Poverty Ratio Under 0.50 | | ~ -0.0000 | 0.0000 | 0.7958 |
| Tot. Pop. With Income to Poverty Ratio Under 1.00 | | ~ -0.0000 | ~0.0000 | 0.3845 |
| Tot. Pop. with a Bachelor's Degree (Ages 25 and Over) | | 0.0000 | 0.0000 | 0.0296 |
| Tot. Pop. with Master's or Professional School Degree or Doctorate (Ages 25 and Over) | | 0.0000 | 0.0000 | 0.2741 |
| Tot. Pop. with a High School Diploma (Ages 25 and Over) | | 0.0000 | ~0.0000 | 0.0680 |
| Tot. Civilian Veterans with some College Education or Associate's Degree (Ages 25 and Over) | | 0.0000 | 0.0000 | 0.6039 |
| Tot. Civilian Veterans Who Are At Most High School Graduates (Ages 25 and Over) | | 0.0000 | 0.0001 | 0.8027 |
| Tot. Households with a Person Living Alone (Ages 65 and Over) | | 0.0000 | 0.0000 | 0.3977 |
| Tot. Mobile Home Households | | 0.0000 | 0.0000 | 0.0580 |
| Tot. Households with Monthly Owner Costs of At Least 30 Pct. Household Income | | 0.0000 | 0.0000 | 0.0038 |
| Tot. Households with Monthly Owner Costs of At Least 50 Pct. Household Income | | ~ -0.0000 | 0.0000 | 0.6191 |
| Tot. Workers with 60+ Minute Commute Time | | 0.0000 | ~0.0000 | 0.9754 |
| Tot. Workers Taking Public Transportation (>15 Minute Commute Time) | | -0.0001 | 0.0002 | 0.4747 |
| Tot. Workers Taking Taxicab, Motorcycle, Bicycle or Other Means to Work | | 0.0000 | 0.0000 | 0.4087 |
| Tot. Pop. with Medicare Only | | 0.0000 | 0.0000 | 0.6055 |
| Tot. Pop. with Other Health Insurance | | 0.0000 | 0.0000 | 0.3727 |
| Tot. Pop. with Employer-Based and Direct Purchase Health Insurance | | 0.0000 | 0.0000 | 0.3660 |
| Tot. Pop. with Employer-Based Health Insurance | | ~0.0000 | ~0.0000 | 0.8445 |
| Tot. Pop. with Employer-Based and Medicare Health Insurance | | 0.0000 | 0.0000 | 0.3479 |
| Tot. Pop. with Public-Only Health Insurance | | 0.0002 | 0.0001 | 0.1944 |
| Tot. Social Associations | | -0.0018 | 0.0007 | 0.0073 |
| Median Income for Hispanic or Latino Households (Dollars) | | ~0.0000 | ~0.0000 | 0.1449 |
| Pct. of Medicare Beneficiaries with Drug Plan (Per 100 People) | | -0.0023 | 0.0014 | 0.0939 |
| Tot. Nurse Practitioners with NPI (Per 1000 Pop.) | | 0.0750 | 0.0294 | 0.0108 |
| Tot. Physician Assistants with NPI (Per 1000 Pop.) | | -0.0109 | 0.0404 | 0.7878 |
| Pct. Medicaid Eligible Beneficiaries Enrolled in a Medicare Advantage Plan (Per 100 Pop.) | | -0.0015 | 0.0011 | 0.1854 |
| Pct. of Pop. in Poverty (Aged 0-17, Estimate) | | -0.0001 | 0.0020 | 0.9571 |
| Tot. Pop. in Poverty (Aged 0-17, Estimate) | | ~0.0000 | ~0.0000 | 0.0003 |
| Pct. Medicaid Eligible Medicare FFS Beneficiaries (Eligible For At Least One Month) | | -0.0011 | 0.0012 | 0.3524 |
| Min Distance to Nearest Medical-Surgical ICU (Miles) | | 0.0011 | 0.0023 | 0.6306 |
| Max Distance to Nearest Health Clinic (FQHC, RHC) (Miles) | | -0.0003 | 0.0014 | 0.8015 |
| Tot. Hospices (Per 1000 Pop.) | | 1.1780 | 0.3702 | 0.0015 |
| Tot. Beds in Long-Term Hospitals (Per 1000 Pop.) | | -0.0084 | 0.0077 | 0.2724 |
| Tot. Short-Term General Hospitals (Per 1000 Pop.) | | -0.2697 | 0.4289 | 0.5294 |
| Tot. Non-Federal General Preventive Medicine Specialists | | 0.0019 | 0.0011 | 0.1024 |
| Tot. Non-Federal MDs | | 0.0000 | 0.0000 | 0.3531 |
| Tot. Medical Specialists | | ~0.0000 | 0.0001 | 0.9281 |
| Tot. Supermarkets and Other Grocery (Except Convenience) Stores (Per 1000 Pop.) | | -0.0323 | 0.0931 | 0.7286 |
| Pct. of Pop. Uninsured (138%-400% Poverty, Under 65 Years Old, Estimate) | | 0.0089 | 0.0023 | 0.0001 |
| Pct. Pop. Lacking Adequate Food Access | | 0.0002 | 0.0030 | 0.9465 |
| Pct. of Adults Who Are Current Smokers | | 0.0079 | 0.0043 | 0.0629 |
| Tot. Food Distribution Program on Indian Reservations (FDPIR) Sites | | -0.0074 | 0.0046 | 0.1072 |
| Min Distance to Nearest Urgent Care (Miles) | | 0.0004 | 0.0014 | 0.7780 |
| Max Distance to Nearest Urgent Care (Miles) | | 0.0003 | 0.0010 | 0.7733 |
|  | |  |  |  |

Figure A.1.

Calibration curve for the logistic regression model.
